# Impact of a school-based water, sanitation and hygiene programme on children’s independent handwashing and toothbrushing habits: a cluster-randomised trial

**DOI:** 10.1007/s00038-020-01514-z

**Published:** 2020-11-03

**Authors:** Denise Duijster, Helen Buxton, Habib Benzian, Jed Dimaisip-Nabuab, Bella Monse, Catherine Volgenant, Robert Dreibelbis

**Affiliations:** 1 Department of Social Dentistry, Academic Centre for Dentistry Amsterdam, University of Amsterdam and VU Universiteit Amsterdam, Amsterdam, The Netherlands; 2 Disease Control Department, School of Hygiene and Tropical Medicine London, London, UK; 3 Department of Epidemiology and Health Promotion, WHO Collaborating Center for Quality Improvement and Evidence-based Dentistry, College of Dentistry, New York University, New York, USA; 4 Gesellschaft für Internationale Zusammenarbeit (GIZ), Metro Manila, Philippines; 5 Department of Preventive Dentistry, Academic Centre for Dentistry Amsterdam, University of Amsterdam and VU Universiteit Amsterdam, Amsterdam, The Netherlands

**Keywords:** Handwashing, Toothbrushing, Habit formation, School programme, Children

## Abstract

**Objectives:**

To explore whether a school-based water, sanitation and hygiene programme, which includes group hygiene activities, contributes to the formation of independent handwashing and toothbrushing habits among Filipino children.

**Methods:**

In this cluster-randomised trial, twenty primary schools were randomly allocated to the intervention or control arm. Intervention schools received group handwashing facilities and implemented daily group handwashing and toothbrushing activities. A soap use to toilet event ratio was calculated to measure children’s independent handwashing behaviour after toilet use, and dental plaque accumulation on Monday morning was measured as a proxy indicator for children’s independent toothbrushing behaviour at home.

**Results:**

Four months after implementation, handwashing and toothbrushing behaviours did not significantly differ between intervention and control schools. The mean soap use in intervention schools and control schools was 0.41 g and 0.30 g per toilet event, respectively (*p* = 0.637). Compared to baseline, mean plaque scores reduced by 4.2% and 3.5% in intervention and control schools, respectively (*p* = 0.857).

**Conclusions:**

Although health benefits have been established, school-based group handwashing and toothbrushing may not be sufficient to increase children’s uptake of independent hygiene behaviours.

**Electronic supplementary material:**

The online version of this article (10.1007/s00038-020-01514-z) contains supplementary material, which is available to authorized users.

## Introduction

Children in low- and middle-income countries (LMICs) suffer from a high burden of preventable diseases and hygiene deficiencies are a common determinant. Diarrheal diseases are a major cause of morbidity among school-aged children (Walker and Black 2010) and have the potential to impact on educational attainment and overall well-being. Handwashing with soap (HWWS) is one of the most cost-effective public health interventions (Curtis and Cairncross 2003) and is associated with a 30% reduction in incidence of diarrhoea (Wolf et al. 2018) and 21% reduction in respiratory illness (Aiello et al. 2008). Dental caries, the most prevalent childhood disease worldwide, severely impacts on children’s body constitution and quality of life through infection, pain, disturbed sleep and discomfort (Sheiham 2006; Monse et al. 2012). Twice daily toothbrushing with fluoride toothpaste (TBFT) is associated with a 24% reduction in tooth decay (Marinho et al. 2003; Duijster et al. 2017; Walsh et al. 2019).

Schools have the potential to significantly contribute to the development and practice of HWWS and TBFT behaviours in children. The importance of a supportive school environment to promote hygiene (particularly HWWS) has been recognised globally through inclusion in the Sustainable Development Goals under target 4.A, which aims to achieve ‘access to handwashing facilities with water and soap in all schools’ by 2030 (United Nations Children’s Fund and World Health Organization 2018). School-based interventions targeting hygiene behaviour change are often limited to educating children about health risks associated with poor personal (oral) hygiene, despite evidence that knowledge transfer and awareness raising seldom lead to sustained behaviour change (Stein et al. 2017; Watson et al. 2017).

Empirical evidence on the impact of school-based hygiene interventions in LMICs is inconsistent and scarce. Cluster-randomised trials of handwashing promotion interventions—both with and without accompanying improvements in school water and sanitation infrastructure—in Kenya (Patel et al. 2013; Freeman et al. 2012, 2014; Pickering et al. 2013), Mali (Trinies et al. 2016), China (Bowen et al. 2007), Laos (Chard and Freeman 2018) and Malawi (Mbakaya et al. 2019) have shown mixed effects on health and educational outcomes. These mixed results are often attributed to poor intervention fidelity and/or compliance (Garn et al. 2013, 2017). With regard to oral health, systematic reviews found little evidence for the effectiveness of oral health education alone, yet school-based interventions combining health education with supervised toothbrushing or professional clinical prevention hold promise for reducing dental caries in LMICs (Cooper et al. 2013; Da Silva et al. 2016; Benzian et al. 2017; Duijster et al. 2017).

Group hygiene activities have been used in schools for decades to ensure that HWWS and/or TBFT is taught, practiced and integrated into daily school routines (Deutsche Gesellschaft fur Internationale Zusammenarbeit and UNICEF 2013; Chard and Freeman 2018). Group hygiene activities serve two purposes. First, they facilitate the logistics of daily HWWS/TBFT of large numbers of students through specially designed infrastructure—typically large, multi-user handwashing stations. Second, the habitual performance of group hygiene activities once or twice per day under supervision of a teacher may positively impact on children’s independent hygiene habits. Habits are learned, automatic behaviours that are triggered unconsciously by familiar cues (e.g., the behaviour ‘putting on a seatbelt’ is triggered when getting into a car), which are reinforced through repetition of the behaviour in a stable context (Wood and Neal 2007; Gardner 2012). It is theorised that daily group hygiene activities in school will translate to independent behaviour uptake at critical times in other settings (e.g., washing hands after defecating and before handling foods; brushing teeth before bedtime). However, evidence for these assumptions and the transfer of habits from school activities to the home context is limited. There is one previous study in Laos that evaluated the behavioural impact of school-based group handwashing specifically (Chard and Freeman 2018); they found an increase in children’s individual handwashing behaviour after toilet use, but these improvements were not sustained over the 18-month evaluation period. The transfer of behaviours to the home context was not assessed.

This study explored how a school-based water, sanitation and hygiene (WASH) programme, which includes daily group HWWS and TBFT activities, contributes to the formation of independent HWWS and TBFT habits in children. Specific objectives were to assess the impact of the programme after 4 months on (1) children’s independent handwashing behaviour and soap use after using the toilet in school, and (2) children’s independent toothbrushing behaviour at home.

## Methods

### Design and intervention: the Fit for School Plus study

This study was part of the Fit for School Plus Study: a parallel cluster-randomised controlled trial evaluating the Fit for School (FIT) approach in the Philippines (Buxton et al. 2019). The FIT approach supports Ministries of Education to improve child health and wellbeing through the institutionalisation of WASH in Schools programmes which integrate evidence-based WASH interventions into daily routines of primary schools (Monse et al. 2010). Interventions include the practice of daily group handwashing with soap and toothbrushing with toothpaste (containing 1450 ppm fluoride). Group HWWS and TBFT activities are conducted once a day under supervision of a teacher or a student. Schools receive manuals and a video introducing group hygiene activities in addition to one basic group handwashing facility (HWF) per classroom, accommodating 20 students at the same time, and the provision of consumables for children’s handwashing and toothbrushing. Overall, responsibility for group hygiene activities is with the ‘homeroom teacher’. Depending of the age of the students, the teacher supervises the activity by him or herself or assigns the class student leader to supervise the activity for their peer students. Students generally like taking responsibility in leading the activity similar to leading the flag ceremony or other group activities within school routines. In 2017, an operation and maintenance (O&M) package was developed based on the FIT principles to improve the usability and cleanliness of school toilets (FIT ‘Plus’). In the Philippines, the Department of Education has been integrating the FIT approach into its national WASH in Schools policy, with technical support from Deutsche Gesellschaft für Internationale Zusammenarbeit (GIZ) Regional FIT Programme.

The FIT Plus study was designed to explore the impact of the FIT ‘Plus’ approach on toilet usability, student and teacher satisfaction with toilet facilities, and children’s HWWS and TBFT behaviour. In this study, the intervention was delivered at a cluster level and the unit of randomisation and analysis was the school. Details on the design of the study and results on toilet usability and satisfaction are described elsewhere (Buxton et al. 2019). Twenty public elementary schools in the Batangas province of the Philippines were randomly selected using the following inclusion criteria: 200–999 students per school, accessible and secure location (within 2 h from Batangas city centre), access to water source, at least one in-use toilet and HWF and a least one multi-story building. The sample was limited to 20 schools due to resource restrictions. The research coordinator generated a random number in MS Excel for the each of the selected schools. Based on the order of ascension schools were allocated to either the control arm (lowest numbers) or the intervention arm (highest numbers) with an allocation ratio of 1:1. No matching or stratification was used.

Schools in the intervention arm were actively supported to improve WASH conditions through: provision of ready to install group HWFs to facilitate daily group HWWS and TBFT; a monthly supply of soap and toothpaste and provision of a toilet O&M package. The O&M package included technical support and supplies (e.g., cleaning rotas, toilet user kits, cleaning products and a manual) to improve the quality of school latrines. In addition to the group activities, children’s behavioural uptake was targeted through provision of stickers to be displayed near to toilets and HWFs designed to cue target behaviour such as handwashing with soap after using the toilet.

Schools in the control arm were informed about the recently released WASH in School policy, which includes promotion of daily group hygiene activities—although no hardware or consumables were provided to participating schools.

### Data collection

Baseline data on handwashing and toothbrushing behaviour were collected 2 weeks prior to the implementation of the FIT Plus approach in July 2017, and endline data were collected 4 months later in November 2017. Process indicators on fidelity and compliance were measured at endline only.

#### Handwashing behaviour

Handwashing facilities in toilets were usually located behind closed doors in the toilet block where toilet cubicles were located, so direct observation of HWWS after using the toilet was not possible. Instead, a soap use to toilet event ratio was calculated as a proxy indicator for HWWS, based on the mass of soap used divided by the number of toilet events per handwashing facility over a 1 day period. In the evening prior to data collection day, new generic soap bars were placed at all locations in toilet facilities (in both intervention and control schools) where it was possible for children to wash hands. Soap bars were weighed accurate to 0.01 g and coded before placement. At the end of the data collection day, soap bars were collected, allowed to dry for 5 days, and weighed again. Soap use was defined as changes in soap mass between the two measurements. The examiners weighing the soap bars (HB and JDN) were blinded to intervention allocation.

The number of toilet events was counted on the day of data collection using bi-directional infrared motion sensors, which counted the number of times a person entered the toilet through the door (toilet event). The soap-use ratio per toilet event was determined by dividing the changes in soap mass by the total number of toilet events. Where toilets facilities were arranged in blocks with multiple toilets cubicles and multiple HWFs inside the block, motion sensors were placed across the entry to the block and the mass of all soaps in the block before use and after drying were summed, and then divided by the total number of toilet events in that block. Data were collected at both baseline and endline; however, motion sensors at baseline were overly sensitive resulting in extremely high toilet event counts. Improved devices (Bi-directional people counter PRx20D1—PTx20-1, Sensor Development International, The Netherlands) that were thoroughly tested were used at endline and baseline data were not used in the analysis.

#### Toothbrushing behaviour

Dental plaque accumulation on the labial surfaces of the upper and lower incisors was assessed on Monday mornings. The amount of dental plaque present served as a proxy indicator for children’s independent toothbrushing behaviour at home between the last toothbrushing activity at school on Friday and the assessment on Monday morning. Ten schools (five in each arm) were randomly selected for data collection. In each of the ten schools, a random sample of 50 children from Grade 2 to Grade 6 classes were selected at baseline and at endline. Children from Grade 1 classes (age 6–7-years-old) were excluded from sample selection, because a large proportion of these children have missing front teeth during the period of mixed dentition. A power calculation for the TBFT outcome indicated that a sample of size of 50 children in each school (5 intervention and 5 control schools) with an intracluster correlation coefficient (ICC) value of 0.01 and a pooled standard deviation of 0.20 was sufficient for a minimum detectable difference of 0.11 assuming an alpha of 0.05 and 90% power.

Data were collected at each school on a Monday morning before any group toothbrushing had taken place. An interview-administered questionnaire was used to collect information on the child’s age, sex, availability of a toothbrush and toothpaste at home and self-reported toothbrushing behaviour, including frequency and last time of toothbrushing. Then, children were instructed to chew on a disclosing tablet (Mira-2-Ton, Hager & Werken, Duisburg, Germany) and to swivel the saliva-disclosing solution in their mouth for 20 s, spit out and rinse once. Lip and cheek retractors were used to allow an unobstructed view of the anterior teeth, so that a digital photograph could be taken of the teeth in an end-to-end position using a smartphone camera (iPhone 5S) with a ring flash. All images received an anonymous ID-code.

The coded images were manually scored using the modified Quigley and Hein plaque index (Paraskevas et al. 2007). Dental plaque accumulation was assessed on the vestibular surfaces of anterior teeth in the upper and lower jaw (central and lateral incisors). For each of the eight incisors, dental plaque was scored on the distal, buccal and mesial surfaces of each tooth on a 6-point scale from no plaque (score ‘0’) to more than 2/3^rd^ of surface covered with plaque (score ‘5’). A mean plaque score for each child was computed by calculating the sum of scores divided by the total number of scored tooth surfaces. All images were scored by one trained and calibrated examiner (DD), and 10% of the images were scored again by a calibrated second examiner (CV) to assess the inter-rater reliability. The weighted percentage agreement between the two examiners was 93.2% and the weighted kappa was 0.73. Both examiners did the scoring at a university in Europe, were not involved in the study execution and were blind to whether the image was taken in control or intervention schools or at baseline or endline.

#### Process indicators

Fidelity and compliance data were drawn from interviews with school principals, from school observations (presence of functioning HWFs and soap availability) and from additional questions that were included in an interview-administered toilet-satisfaction survey among children (the practice of group hygiene activities in schools, such as the last time children washed their hands or brushed their teeth as a class) (Buxton et al. 2019). The data were collected once at endline by trained study staff. In each school, a sample of 16 children from grade 4 and above were randomly selected for the toilet-satisfaction survey (320 children in total). Data were collected using handheld digital devices running Open Data Kit software. School principals and surveyed children could not be blinded to intervention allocation.

### Data analysis

Data were analysed using STATA v.15 (Stata Corp, College Station, Texas, USA). For HWWS-related data, the unit of analysis was the toilet, clustered by school. Generalised estimating equations (GEE) were used to provide population-averaged effects and to adjust for school-level clustering. The mean average soap use per toilet event was calculated per school, and p-values are reported to indicate differences between intervention and control schools. For TBFT-related data, children were the primary unit of analysis. GEE was performed using a difference in difference approach, which provides the mean difference in plaque scores in the intervention group compared to the control group, after adjusting for differences at baseline. The analysis was adjusted for age and sex, and clustered by school.

Two analyses were conducted; a per protocol analysis including all intervention schools, and a second sub-analysis in schools where hygiene activities were regularly implemented, defined as those intervention schools where at least 50% of children interviewed reported that ‘group handwashing and toothbrushing was practiced today or the last school day’. The statistical analyses were not performed blind.

## Results

**Fig. 1:**
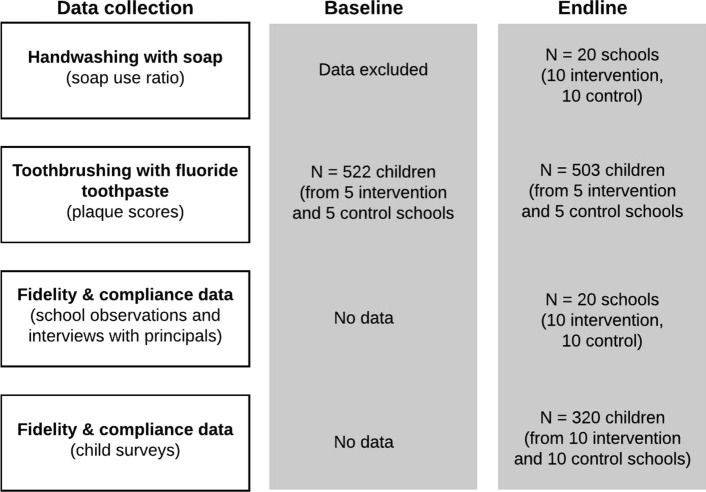
Diagram of the number of units (clusters, participants) for which data were available at baseline and at endline in the analysis

Figure 1 shows a diagram of the number of units (clusters or participants) at baseline and endline for which data were available in the analysis.

### Fidelity and compliance

The average number of children per school was 420 in intervention schools and 449 in control schools. All schools were from the same peri-urban environment, and age distribution of students was similar in the intervention and control arm. More information about baseline characteristics of schools can be found in Buxton et al. (2019). All intervention schools received components to assemble the group HWFs in the first month of the intervention (August); however, only half of the intervention schools had a group HWF ready for use by September. By endline, all intervention schools had assembled at least one group HWF (Table 1). At endline, soap was twice as likely to be available at handwashing facilities in or immediately outside of toilet cubicles in the intervention group than in the control group (RR: 2.02 (*p* < 0.001)). Intervention-provided stickers to cue children’s independent HWWS had been displayed in 50% of toilets.

**Table 1:** Process indicators of the schools, Fit for School Plus study, Philippines, 2017

School observations (at endline)	Control schools	Intervention schools	p*
Percentage of toilets with water available at a facility to wash hands after toilet use			
Baseline	77%	80%	RR: 1.068 *p* = 0.297
Endline	76%	82%	
Percentage of handwashing facilities with soap available			
Baseline	27%	37%	RR: 2.019 *p* < 0.001
Endline	38%	54%	
Percentage of toilets with stickers to HWWS on display at endline	0%	50%	< 0.001
Reported data from school principals			
Number of schools with at least one group handwashing facility	0/10	9/10	< 0.001
Number of schools that report daily group HWWS	4/10	7/9	< 0.001
Number of schools that report daily group TBFT	3/10	8/9	< 0.001
Percentage of children that reported that group handwashing			
Was ever practiced	23%	75%	< 0.001
Was practiced today or the last school day	15%	62%	< 0.001
Is practiced daily	10%	50%	< 0.001
Is practiced before eating	14%	27%	0.004
Is practiced with soap	26%	75%	< 0.001
Percentage of children that reported that group toothbrushing			
Was ever practiced	20%	85%	< 0.001
Was practiced today or the last school day	10%	72%	< 0.001
Is practiced daily	9%	64%	< 0.001
Is practiced after eating	8%	73%	< 0.001
Is practiced with toothpaste	20%	85%	< 0.001

*Chi-square test

Data from surveys with both school principals and children indicate that group HWWS and TBFT were not happening on a daily basis in all the intervention schools, and that some control schools were conducting the group activities independently (Table 1). Participation in group HWWS in the last 24 h was reported by 62% of children in intervention schools compared to 15% of children in control schools (*p* < 0.001). Reported TBFT in the last 24 h was 72% in intervention schools compared to 10% in control schools (*p* < 0.001).

### Handwashing with soap after toilet use

Data for the soap use to toilet event ratio were only available at endline. On average, there was a 0.41 g (standard deviation (SD) = 1.56) reduction in soap mass for every toilet event in intervention schools. In control schools, a mean of 0.30 g (SD = 0.86) was used per toilet event (Table 2). This difference was not statistically significant (*p* = 0.637). Similar patterns were observed when analysis was stratified to classroom and non-classroom toilets. Further analysis was conducted to exclude intervention schools that did not regularly implement the group hygiene activities (n = 2), but no significant difference were observed (Table 2).

**Table 2:** Difference in soap-use ratio between intervention and control schools, Fit for School Plus study, Philippines, 2017

	All schools			Only including intervention schools where at least 50% of children interviewed reported that ‘group handwashing and toothbrushing was practiced today or the last school day’		
	Control mean ± SD	Intervention mean ± SD	*p* value*	Control mean ± SD	Intervention mean ± SD	*p*-value*
All toilets	0.30 g ± 0.86/event	0.41 g ± 1.56/event	*p* = 0.637	0.30 g ± 0.86/event	0.52 ± 1.83/event	*p* = 0.458
Non-classroom toilets	0.13 g ± 0.20/event	0.24 g ± 0.66/event	*p* = 0.423	0.13 g ± 0.2/event	0.32 g ± 0.8/event	*p* = 0.301
Classroom toilets	0.39 g ± 1.05/event	0.54 g ± 2.00/event	*p* = 0.689	0.39 g ± 1.05/event	0.65 ± 2.29/event	*p* = 0.578

*Generalised estimating equations model, soap use ratios clustered by study group

### Toothbrushing at home over the weekend

Dental plaque measurements were collected from 522 children at baseline and from 503 children at endline. All children, apart from 6, reported to have a toothbrush and toothpaste at home. At baseline, the mean dental plaque score of children was 3.36 (SD = 0.97) in intervention schools and 3.39 (SD = 0.95) in control schools (Table 3). This corresponds with an average of 1/3^rd^ of all tooth surfaces covered with dental plaque. Four months after implementation of the FIT Plus intervention, mean plaque scores reduced by 0.14 (4.2%) in intervention schools and 0.12 (3.5%) in control schools. This difference was not statistically significant, also after adjustment for baseline differences, age, sex and clustering (*p* = 0.857). Similar results were found when intervention schools that did not regularly conduct hygiene activities were excluded from the analysis (Table 3). The majority of children reported having brushed their teeth on the morning of data collection, and children generally reported a high toothbrushing frequency without significant differences between intervention and control schools; yet, toothbrushing activity was not reflected in their high dental plaque scores (see Table 4).

**Table 3:** Difference in dental plaque accumulation between intervention and control schools, Fit for School Plus study, Philippines, 2017

Dental plaque scores	All schools				Only including intervention schools where at least 50% of children interviewed reported that ‘group handwashing and toothbrushing was practiced today or the last school day’			
	Baseline mean ± SD	Endline mean ± SD	Difference	DID mean difference* β (95% CI), *p*-value	Baseline mean ± SD	Endline mean ± SD	Difference	DID mean difference* β (95% CI), *p*-value
Control	3.39 ± 0.95	3.27 ± 0.96	− 0.12	β = 0.02 (− 0.21; 0.25) *p* = 0.857	3.39 ± 0.95	3.27 ± 0.96	− 0.12	β = 0.01 (− 0.25; 0.26)
Intervention	3.36 ± 0.97	3.22 ± 0.98	− 0.14		3.34 ± 0.95	3.22 ± 0.97	− 0.12	*p* = 0.963

Generalised estimating equations model: *DID* Difference in Difference analysis

Primary teeth were excluded from the analysis

*Adjusted for age, sex and school-level clustering

**Table 4:** Differences in self-reported toothbrushing behaviour between intervention and control schools, and correspondence with dental plaque scores at endline, Fit for School Plus study, Philippines, 2017

	*n* (%)	Mean corresponding plaque score
Toothbrushing frequency	Control schools	
Once a day	20 (8.2%)	3.33
Twice a day	87 (35.8%)	3.29
Three times a day	122 (50.2%)	3.21
More than three times a day	13 (5.4%)	3.35
	Intervention schools	
Once a day	36 (14.2%)	3.19
Twice a day	76 (30.0%)	3.41
Three times a day	133 (52.6%)	3.11
More than three times a day	8 (3.2%)	3.14
*p*-value (difference in toothbrushing frequency between intervention and control schools)	*p* = 0.100	
Last time children brushed	Control schools	
This morning	188 (75.8%)	3.11
Yesterday	57 (23.0%)	3.74
Two days ago or more	3 (1.2%)	3.92
	Intervention schools	
This morning	199 (78.4%)	3.13
Yesterday	52 (20.5%)	3.53
Two days ago or more	3 (1.2%)	3.15
*p*-value (difference in last time brushed between intervention and control schools)	*p* = 0.790	

*Chi-square test

## Discussion

HWWS and TBFT are recognised globally as highly effective hygiene activities preventing infectious diseases and tooth decay; both activities are able to deliver positive health benefits when practiced in schools (Monse et al. 2010; Duijster et al. 2017; McGuinness et al. 2018). Yet, this study did not show an impact of school-based HWWS and TBFT on proxy measures of children’s independent hygiene habits outside of organised group activities at school, as no significant differences in soap use to toilet event ratio and dental plaque were found between intervention and control schools.

For HWWS, children in intervention schools used an average of 0.41 g of soap per toileting event compared to 0.30 g used by children in control schools. Although these ratios do not significantly differ, they may indicate that a large number of children in both the intervention and control groups used soap after toileting. Previous studies have shown increases in HWWS in schools when materials for handwashing (water and soap) are provided at a single location (Dreibelbis et al. 2016). Yet, some limitations should be considered in the interpretation of our findings related to HWWS. Measuring behaviour through observation is challenging due to a possible impact of the Hawthorne effect or social desirability bias (McCambridge et al. 2014). For HWWS, it was attempted to avoid this by using sensors rather than human observers. In order to desensitize children to the presence of sensors, inactive ‘dummy’ sensors were installed approximately 1–2 weeks in advance of data collection and replaced with the real sensors on the day of data collection. Despite this measure, a certain amount of wilful interaction with the sensors cannot be fully ruled out, which may have resulted in over-estimates of toileting events. According to the European Chemicals Bureau (EU TGD 2003) and Comiskey et al. (2017), adults use an average of 0.8 (0.5–1.1) g of solid bar soap per handwashing event. However, direct extrapolation of soap-use ratios from our study to estimates of handwashing behaviour could over-estimate actual hygiene practices. The presence of new soap bars used for this study caused some excitement among children; data collectors observed children using soap for multiple purposes other than washing hands, such as washing faces, clothes, toilet walls or even rugs. Excitement was much higher in control schools, since soap had already been available in the handwashing stations in intervention schools during the full study period, while children in control schools were only exposed to new, pleasant smelling soap on the day of data collection. This may explain the relatively high soap consumption per toilet event in control schools. Another limitation was that baseline data for HWWS had to be excluded from the analysis due to overly sensitive event counts. Therefore, the analysis could not be adjusted for potential differences in soap use between intervention and control schools at baseline, and no conclusions about changes in soap use during the 4 month study period could be drawn.

For TBFT, the findings of generally high plaque scores indicate that toothbrushing is not an established behaviour in the home context. School-based toothbrushing in intervention schools did not lead to increased rates of children’s independent TBFT behaviour at home. The FIT intervention aims to provide the necessary conditions for hygiene habit formation in the school setting, including the creation of a supportive environment through daily-repeated group activities and provision the necessary materials. However, neither the programme nor the study provided any intervention for the home setting. Furthermore, the availability of basic requirements (toothbrush and toothpaste) to execute the new behaviour was not assured, which might have been the limited factor for children to develop independent toothbrushing behaviour at home. Nearly all children reported the availability of a toothbrush and toothpaste at home, but this information should be treated with caution. Children in all public schools in the Philippines have received oral health education from Grade 1 to Grade 6, leading to knowledge about the appropriate behaviour. As children may tend to give socially desirable answers in interviews, the information may not be reliable. Our novel method of analysing digital plaque images taken on Monday to measure oral hygiene habits at home provides reliable scores related to plaque removal. Our data showed a stark contrast between these measurements and self-reported toothbrushing data. This highlights again that self-reported behaviour information should be interpreted with great caution due to risk of significant bias.

There are a few methodological limitations of this study that should be acknowledged. This study describes real-life implementation research which has associated challenges with programme compliance. Process data revealed delays in the construction of group HWFs and surveys with children indicated that schools had not achieved 100% coverage of daily group HWWS and TBTF activities. Challenges with intervention compliance are common barriers to effective school-based hygiene interventions (Garn et al. 2013, 2017) and limits the ability of potentially effective interventions to reach their full potential. Unplanned crossover posed another challenge in our study; some control schools started to implement group hygiene activities independently of the study due to orientation of school principals on the new WASH in Schools policy of the Department of Education. This may provide an explanation for the lack of significant impact in this study. The length of the intervention (mid-August to mid-November) may have also been too short a period to impact significantly on habit formation. The study by Chard and Freeman (2018), which evaluated the impact of a comprehensive WASH in Schools programme—including daily group handwashing—in Laos, found that schools required 6–12 months after programme implementation to establish group handwashing. Children attending schools where group handwashing was conducted were more likely to practice individual HWWS after toilet use, but not until 6–18 months after programme implementation and improvements were not sustained. They concluded that ‘complementary strategies need to be concurrently promoted for effective and sustained individual HWWS at critical times’.

Findings of this study contribute to a rich body of literature on hygiene interventions. Promoting hygiene behaviour change is challenging, and evidence from both successful and unsuccessful interventions are highly relevant to gain a proper understanding of what elements contribute to effective behaviour change strategies, and what do not. A few studies have shown positive effects of using ‘nudges’ in handwashing promotion interventions, ranging from visual cues such as posters and stickers to innovate nudges such as brightly coloured HWFs and coloured paths with painted footprints and arrows leading from toilets to HWFs (Neal et al. 2015; Grover et al. 2018). There is some evidence for interventions that create a supportive environment; a review showed that provision of access to and convenience of handwashing materials significantly improved the practice of HWWS (Curtis et al. 2009), and distribution of free fluoride toothpaste and brushes significantly reduced caries rates in high risk children living in deprived areas (Davies et al. 2002).

Our study’s findings do not diminish the relevance of improving both hand and oral hygiene behaviours through multiple strategies in schools. Both HWWS and TWFT have strong evidence for positive health outcomes in children and performing them as a group in schools may be a feasible way to manage the logistics of establishing hygiene routines that involve large number of children. In particular, the health benefits of school-based fluoride toothbrushing have been firmly established, with research showing a 24% (18–38%) reduction in caries increment (Duijster et al. 2017). As children are not performing this highly effective intervention at home, it is of utmost public health interest to institutionalise the habit in the school context. At least while attending school and participating in school-based HWWS and TBFT activities, children have the opportunity to benefit from these interventions. How group handwashing activities can complement or leverage other intervention strategies for improving HWWS requires further investigation.

### Conclusion

Findings from this study did not identify evidence that school-based group handwashing increases individual handwashing behaviour after 4 months. The study findings also suggest that school-based group toothbrushing activities will not automatically develop into independent toothbrushing behaviours of children in the home environment. The interventions were limited to the school environment and activities and provision of toothbrushes and toothpaste in the home context were not part of the intervention. Behaviour transfer of school-based hygiene activities to the home context does not happen automatically and requires effective mechanisms for behaviour change at the household level to ensure habit formation and sustainability. Group hygiene activities should be considered a component of, rather than the exclusive focus, of school-based HWWS and TBFT interventions. Future research is required to understand how group activities can inform independent habit formation.

## Electronic supplementary material

Below is the link to the electronic supplementary material.

Supplementary material 1 (DOC 217 kb)
